# The Role of Fear of Progression in Psychosocial Adjustment in Middle-Aged and Older Women with Oncological Disease: A Path Analysis

**DOI:** 10.3390/jcm15062234

**Published:** 2026-03-15

**Authors:** Sandra Silva, Ana Bártolo, Débora Paiva, Isabel M. Santos, Sara Monteiro

**Affiliations:** 1 RISE-Health, Department of Education and Psychology, University of Aveiro, 3810-193 Aveiro, Portugal; smonteiro@ua.pt; 2 RISE-Health@UPT, Portucalense University, 4200-072 Porto, Portugal; ana.bartolo@upt.pt; 3 Department of Education and Psychology, University of Aveiro, 3810-193 Aveiro, Portugal; debmpaiva695@gmail.com; 4 William James Center for Research, Department of Education and Psychology, University of Aveiro, 3810-193 Aveiro, Portugal; isabel.santos@ua.pt; 5 Department of Social Sciences and Management, Universidade Aberta, 1269-001 Lisbon, Portugal; 6 Center for Global Studies, Universidade Aberta, 1269-001 Lisbon, Portugal

**Keywords:** fear of progression (FoP), psychosocial adjustment, oncological disease, mediating effect

## Abstract

**Background/Objectives**: The global prevalence of cancer is increasing, with 50% of new diagnoses occurring in people over 65 years old. Despite this demographic trend, psychosocial research has focused largely on younger populations, leaving a limited understanding of how middle-aged and older adults adjust to cancer. Fear of progression (FoP) has emerged as a key component of emotional vulnerability in cancer survivors, yet its role in shaping psychological distress and health-related quality of life (HQoL) in these age groups remains understudied. This study aimed to examine psychosocial adjustment in middle-aged and older women with cancer and to test the mediating role of FoP in the relationship between emotional distress, post-traumatic stress and HQoL. **Methods**: The sample consisted of 135 women aged 45 to 79 years (M = 52.94; SD = 6.58), of whom 80.7% (*n* = 109) were middle-aged (45–59 years) and 19.3% (*n* = 26) were older (60–79 years). In addition to a clinical and sociodemographic questionnaire, the following self-report measures were used: Fear of Progression Questionnaire—Short Form (FoP-Q-SF), Hospital Anxiety and Depression Scale (HADS), Post-Traumatic Stress Disorder Checklist—Civilian Version (PCL-C), and the European Organization for Research and Treatment of Cancer Quality of Life Questionnaire Core-30 (EORTC QLQ-C30). Mann–Whitney tests were used to explore age-group differences as part of the sample characterization. Correlational analyses examined associations among FoP, anxiety and depression, post-traumatic stress, and HQoL. The mediating role of FoP was tested using path analysis with bias-corrected percentile bootstrapping. **Results**: Older women reported slightly higher levels of depression and poorer physical and social functioning compared to middle-aged women, but with a small effect size (0.2). Path analysis revealed a significant indirect effect of FoP on the relationship between post-traumatic stress and HqoL (indirect effect 1; estimated effect = −0.174; 95% CI [−0.293, −0.084]; *p* < 0.001), as well as between depression and HqoL (indirect effect 2; estimated effect = −0.181; 95% CI [−0.301, −0.086]; *p* < 0.001). **Conclusions**: Middle-aged and older women with cancer showed broadly similar psychosocial profiles. FoP emerged as a central mechanism linking psychological distress to poorer HQoL across age groups. Interventions targeting FoP may therefore be beneficial for improving psychosocial adjustment in women living with cancer, regardless of age.

## 1. Introduction

By the year 2045, it is projected that the incidence of new cancer cases will increase by approximately 64%, amounting to 32.6 million new diagnoses and 16.9 million deaths annually. Of these new cases, approximately 50% are expected to occur in individuals aged 65 and older, with over 60% of cancer-related fatalities occurring within this age group [1]. This demographic trend highlights aging as a major risk factor for cancer. It underscores the need to better understand the psychosocial impact of the disease across the later stages of adulthood.

Despite this epidemiological reality, research involving adults in midlife and older age remains limited. This gap is also evident in social and behavioral research, which has traditionally focused on younger populations, leaving an insufficient understanding of how cancer affects the quality of life and mental health in older age groups [2,3,4,5]. Several factors contribute to this underrepresentation, including comorbidities (dementia and cardiovascular disease), polypharmacy, poor nutrition, unhealthy lifestyle habits (smoking, alcohol dependence, and sedentary lifestyle), loss of autonomy in daily activities, and social isolation. All of these factors make it difficult to differentiate between physical and psychological symptoms resulting from cancer and other manifestations commonly associated with aging or other morbid conditions [6].

Given the scarcity of evidence, it becomes imperative to consider the psychosocial challenges inherent in the cancer trajectory. A cancer diagnosis is a disruptive event, still deeply associated with life-threatening situations. This risk is compounded by the severe side effects of treatments, giving the diagnosis a traumatic and stressful nature capable of triggering diverse cognitive and emotional reactions—from adjustment difficulties to anxiety, depression, or Post-Traumatic Stress Disorder (PTSD) [7,8,9]. Although depression and anxiety affect between 10% and 20% of cancer patients in general [10,11,12], the literature involving elderly patients indicates depression rates between 17% and 26% and anxiety rates between 2.5% and 23%, depending on the tumor type, therapeutic phase, and diagnostic methodology [13,14]. Regarding PSPT, a meta-analysis estimates a lifetime prevalence of 12.6% [15], while other studies suggest that 10% to 20% of patients may present with subsyndromal levels [16], reinforcing the importance of investigating vulnerability factors in different age cohorts.

In addition to these difficulties, fear of disease progression (FoP—a persistent and realistic fear that the disease will progress or may relapse) [17], although less studied, has proven to be prevalent and relevant at various stages of the disease—from diagnosis and treatment to remission. Luigjes-Huizer and colleagues [18] found that 88% of young people (18–29 years old) in remission from the disease reported a clinically relevant level of FoP, and 48% of young people reported a severe level of FoP requiring intervention. Among older adults (≥75 years) in remission, 37% reported clinically relevant FoP and 9% reported severe FoP, with a similar pattern observed in patients with active disease. In turn, Lim and Humphris [19] also reported an inverse relationship between age and FoP, noting that the strength of the association has decreased in the last decade, with less disparity between FoP levels of younger and older patients. Importantly, evidence from a broader age range (18–85 years) also shows a direct relationship between FoP and symptoms of depression and anxiety [20], suggesting that FoP may reflect a more general vulnerability process that cuts across age groups rather than a phenomenon restricted to specific developmental stages. Taken together, these findings highlight FoP as a central component of emotional vulnerability in cancer survivors, raising important questions about how this fear may influence broader indicators of health-related quality of life (HQoL).

While the negative impact of depression [21], anxiety [22], and/or FoP [23] on therapeutic adherence and QoL [24] is recognized, possibly affecting treatment outcomes [25], length of hospital stay [26], recovery expectations [27], and mortality rates [28,29], research focused on the middle-aged and elderly population is still limited. This research gap is also especially relevant if we consider the evolution of the concept of quality of life, which, as it has become more comprehensive, has come to encompass not only physical health, but also mental health, social relationships, and satisfaction with one’s own life [30]. This broader perspective is crucial, especially for middle and older adults, where maintaining quality of life is often the main goal of treatment, even surpassing the priority of increasing lifespan [31].

Within this broader context, it is also important to recognize that the experience of cancer can differ substantially between the sexes. The scientific literature underscores that the cancer experience is profoundly influenced by gender, as social expectations and socialization patterns shape how individuals perceive and react to the disease. According to Social Role Theory, women face idiosyncratic challenges stemming from the historically assigned role of “caregiver.” This social construct dictates not only greater permeability to emotional expression but also additional pressure to maintain family and domestic responsibilities, even during treatment. Consequently, the female experience of the disease is marked by a complex intersection between physical vulnerability and the maintenance of supportive roles, generating patterns of distress and coping strategies qualitatively distinct from those observed in men [32].

Furthermore, middle-aged and elderly women constitute a particularly relevant group for psychosocial research. They are disproportionately affected by certain types of cancer (e.g., breast and gynecological cancer) [33] and often face unique challenges related to body image [34]. Therefore, focusing the study exclusively on women (middle-aged and elderly) allows for a more precise and sensitive understanding of psychosocial adjustment, while reducing the heterogeneity that typically complicates studies with participants of both sexes.

In the present study, the decision to focus exclusively on women was primarily methodological and not based on the expectation that FoP, psychological distress, or quality of life operate differently by gender. Accordingly, gender was not conceptualized as a variable within the analytic model, but rather as an inclusion criterion aimed at reducing the heterogeneity associated with gender-specific patterns in emotional expression, caregiving roles, and cancer types. This approach allowed for a more precise examination of the mechanisms under investigation.

### Research Aims and Hypotheses

In this context, the present study pursued two complementary aims. First, we conducted an exploratory characterization of middle-aged and older women to examine whether these groups differed in depression, anxiety, post-traumatic stress, fear of progression, and health-related quality of life. These comparisons were not conceptualized as a primary objective, nor were directional hypotheses formulated, given the lack of consistent theoretical expectations regarding age. Rather, age was treated as a descriptive variable to contextualize the sample, while the central focus of the study lay in understanding the mechanisms linking fear of progression, psychological distress, post-traumatic stress, and health-related quality of life—mechanisms that may operate similarly across later adulthood (45+). Therefore, the central objective of this research was to test whether fear of disease progression acts as an explanatory (mediating) mechanism in the relationship between psychological distress (depression and post-traumatic stress) and HRQoL. In accordance with the adopted theoretical framework, the following hypotheses were formulated:

**H1.**  *Higher levels of depression would be associated with poorer health-related quality of life through higher fear of progression.*

**H2.**  *Higher levels of post-traumatic stress would be associated with poorer health-related quality of life through higher fear of progression.*

## 2. Materials and Methods

### 2.1. Participants and Procedure

This study employed a cross-sectional design, using a convenience sample recruited through online platforms. For the following participants, volunteers had to meet the following criteria: (i) be 45 years of age or older, (ii) be female, and (iii) have had a cancer diagnosis for at least six months. The research received approval from the data protection officer and the Ethics and Deontology Committee of the University of Aveiro (ethics approval number 19-CED/2021). All procedures respected the norms of the Portuguese Psychologists’ Code of Ethics, the Declaration of Helsinki, and the Oviedo Convention. Furthermore, the study complied with the General Data Protection Regulation (GDPR), guaranteeing anonymity and confidential treatment of all collected information.

For data collection, the LimeSurvey platform, hosted on the University of Aveiro’s servers, was used. The recruitment strategy was based on the digital dissemination of the questionnaire: invitations were sent by email to various higher education institutions and patient associations. Simultaneously, in order to diversify the sample, the study was shared in support communities for cancer patients on the social networks Facebook and Instagram. Recruitment began in September 2023 and ended in July 2024, when the number of responses stabilized. In line with developmental research, adults aged 45 and older are often conceptualized as entering later adulthood, a period characterized by increased health vulnerability and shifting social roles. This framework guided the age categorization used in the present study [35].

### 2.2. Measures

#### 2.2.1. Sociodemographic and Clinical Questionnaire

The participants were characterized using a questionnaire developed for this study, designed to collect sociodemographic data and clinical indicators. In addition to the sociodemographic aspect, the instrument assessed variables such as age, sex, education level, professional status, marital status, and parenthood. Simultaneously, the questionnaire sought a detailed survey of the participants’ clinical profile, including diagnosis, disease detection data, stage and current stage of the pathology, as well as the presence of metastases. Information regarding therapeutic history was also collected, including completed or ongoing treatments, and the possible existence of psychological support.

#### 2.2.2. Fear of Progression Questionnaire—Short Form (FoP-Q-SF)

Fear of disease progression was assessed using a 12-item instrument, scored on a 5-point Likert scale. The final score, obtained by summing the responses, ranges from 12 to 60 points; the higher the score, the greater the level of fear reported. The version validated for the Portuguese population presents robust psychometric properties, with an internal consistency of 0.86 [36]. In the present sample, the instrument showed high reliability, registering a Cronbach’s alpha of 0.88.

#### 2.2.3. Hospital Anxiety and Depression Scale (HADS)

To assess emotional symptomatology, the Portuguese version of the Hospital Anxiety and Depression Scale (HADS; Portuguese version by Pais-Ribeiro and colleagues, 2007) is used. This instrument consists of 14 items, divided into two subscales of seven items each: one for anxiety and the other for depression. Responses are recorded on a 4-point Likert-type scale (0 to 3), resulting in a total score per domain ranging from 0 to 21. Higher scores reflect higher levels of symptoms. The authors of the Portuguese version found a Cronbach’s alpha of 0.76 for anxiety and 0.81 for depression [37]. In the present study, Cronbach’s alpha for the depression subscale was 0.76, and for the anxiety subscale, it was 0.85.

#### 2.2.4. Post-Traumatic Stress Disorder Checklist—Civilian Version (PCL-C)

The Portuguese version of the PCL-C was used, an instrument that assesses subjective experience in the face of potentially traumatic events [38]. This scale has been widely applied in various contexts, including war veterans, victims of harassment and accidents, at-risk professionals (firefighters and police officers), the elderly, and cancer patients [39]. The inventory consists of 17 items, corresponding to the diagnostic criteria for Post-Traumatic Stress Disorder (PTSD) of the DSM-IV-TR [40]. Responses are recorded on a five-point Likert scale (1 = not at all to 5 = extremely). According to Marcelino and Gonçalves [38], the total score can be obtained by summing the items, which are then divided into dimensions: ‘reliving’ (items 1–5), ‘avoidance’ (6–12), and ‘hyperarousal’ (13–17). The original Portuguese version demonstrated excellent psychometric properties with a Cronbach’s alpha of 0.94 [38], obtaining, in the present sample, a Cronbach’s alpha of 0.95.

#### 2.2.5. The European Organization for Research and Treatment of Cancer Quality of Life Questionnaire Core-30 (EORTC QLQ-C30)

Health-related quality of life was assessed using the EORTC QLQ-C30, in its version validated for the Portuguese population by Pais-Ribeiro and colleagues [41]. This instrument, composed of 30 items, presents a multidimensional structure that integrates five functional scales, the global health status/QoL scale, three symptom scales and single-item measures.

Regarding the EORTC QLQ-C30, only four functional scales were selected: physical function, role performance (or role function), emotional function, and social function, totaling 13 items. These subscales were selected because they represent core domains of functional health-related quality of life, which aligns with the conceptualization of HRQoL adopted in the analytic model. In contrast, the symptom scales and the global health status/QoL item were excluded because they assess conceptually distinct constructs (e.g., symptom burden and overall health perception) that do not correspond to the functional domains targeted in the mediation model. Restricting the measurement to the functional scales therefore ensured conceptual coherence and allowed for a more precise operationalization of the latent HRQoL construct. This approach is consistent with previous research that has also used the QLQ-C30 functioning subscales to model HRQoL as a unified construct.

Responses are recorded on a 4-point Likert-type scale (ranging from ‘not at all’ to ‘very much’), with consecutive brutal responses transformed into a scale of 0 to 100. In these domains, higher scores are indicators of better functioning. Regarding the internal consistency of the Portuguese version, the reported Cronbach’s alpha values were 0.74 for physical function, 0.85 for role performance function, 0.87 for emotional function, and 0.78 for social function [41]. In the sample of the present study, reliability remained robust, with coefficients of 0.77 for physical function, 0.80 for role performance, 0.85 for emotional function, and 0.82 for social function.

### 2.3. Statistical Analysis

Statistical analysis was performed with the Statistical Package for Social Sciences (SPSS), version 28 (IBM Corp., released 2021). The sample characteristics were analyzed using descriptive statistics (percentages, means, standard deviations (SDs) and range). Mann–Whitney tests were used to compare psychosocial variables (depression, anxiety, post-traumatic stress, and FoP) between middle-aged and older women. The differences were considered significant if the *p* value was <0.05. To verify the existence of relationships between fear of progression, psychological distress, post-traumatic stress and quality of life in cancer survivors, correlation analyses were conducted. The role of FoP in psychosocial adjustment to cancer disease was assessed by a path analysis with post-traumatic stress and depression as independent variables, fear of progression as a mediating variable and health-related quality of life as a latent dependent variable. The parameters of the structural equation model were estimated using the maximum likelihood and bootstrap methods to test the corresponding mediating effects.

## 3. Results

### 3.1. Sample Characteristics

The sample included 135 women aged between 45 and 79 years, representing a middle-aged and older adult oncology population. Of these participants, 80.7% (*n* = 109) were middle-aged adults (45–59 years) and 19.3% (*n* = 26) were older adults (60–79 years) (see Table 1). Regarding education, 43.0% of the participants (*n* = 58) had an undergraduate degree, while 19.3% (*n* = 26) had only an elementary education. The majority were married or cohabiting (68.9%, *n* = 93). In terms of employment, 45.2% (*n* = 61) were employed/self-employed, while 18.5% (*n* = 25) were retired, with retirement being higher in older adults (50.0%, *n* = 13). Most participants lived with a spouse/partner (68.1%, *n* = 92), and 60.7% (*n* = 82) lived with their son/daughter. The most common cancer type was breast cancer (77.8%, *n* = 105). Participants had been diagnosed on average 4.24 years prior to study enrolment (SD = 4.02; range 0.58–28.6). At the time of the study, 39.3% (*n* = 53) were still undergoing treatment. Previous treatments included chemotherapy (76.3%, *n* = 103), surgery (73.3%, *n* = 99), hormone therapy (63.7%, *n* = 86), and radiation therapy (61.5%, *n* = 83). Additionally, 37.8% (*n* = 51) of participants reported having used mental health services. The most frequently reported comorbidities were hypertension (13.3%, *n* = 18), high cholesterol (10.4%, *n* = 14), and osteoarthritis (9.6%, *n* = 13).

As an exploratory analysis, psychosocial variables were compared between middle-aged and older women (Table 2). Results indicated that older adults exhibited slightly higher levels of depression and poorer physical and social functioning; however, effect sizes were small, suggesting subtle differences. Additionally, no significant differences were observed in fear of progression, anxiety, and post-traumatic stress.

### 3.2. Correlational Analyses: Relationship Between Fear of Progression, Psychological Distress, Post-Traumatic Stress, and Quality of Life

Complementing the descriptive and exploratory group-based comparisons presented in Section 3.1, correlational analyses were conducted in the total sample using continuous variables (see Table 3). Fear of progression showed a strong positive correlation with anxiety symptoms and the total score of post-traumatic stress. Additionally, there were moderately significant associations between fear of progression and the subdomains of post-traumatic stress, including re-experience, avoidance, and hyperactivation, as well as depressive symptoms. These results suggest that individuals with a greater fear of progression also tend to have higher levels of distress and post-traumatic stress. Fear of progression also demonstrated strong negative correlations with emotional, physical, and social functioning, along with a moderate correlation with role functioning. This indicates that individuals with a greater fear of progression show poorer quality of life indicators, with a significant impact on various domains of functioning. The relationships between anxiety, depression, and functioning were also significant. Anxiety showed a stronger negative relationship with emotional functioning, with moderate correlations with other dimensions of quality of life, while depression was moderately associated with all dimensions. The weakest correlations were found with role functioning. Post-traumatic stress, in turn, showed moderate negative relationships with emotional, physical, and social functioning, as well as moderate positive correlations with anxiety and depression. Importantly, the functional HRQoL subscales showed moderate to strong intercorrelations, indicating substantial shared variance among emotional, physical, social, and role functioning. This pattern supports their treatment as indicators of a single latent HRQoL construct in the mediation model, as theoretically expected and consistent with previous research [42].

As shown in Table 3, age was not significantly associated with any psychosocial variables, except for a weak positive correlation with depression (r = 0.216, *p* < 0.05).

### 3.3. Path Analysis: The Role of Fear of Progression

**Figure 1:**
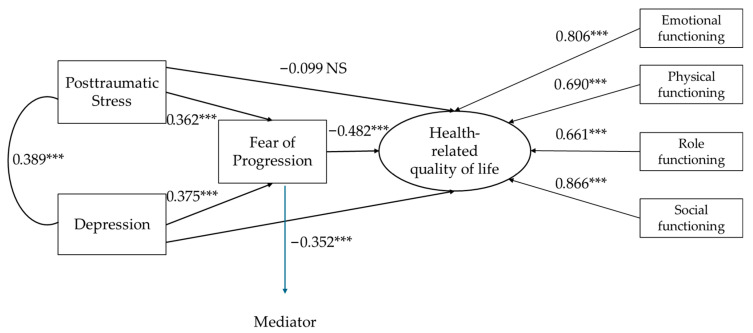
Path analysis: a mediation model. Note. *** *p* < 0.001; NS = non-significant. indirect effect 1: post-traumatic stress → fear of progression → health-related quality of life; indirect effect 2: depression → fear of progression → health-related quality of life.

Based on preliminary correlations, a path analysis was conducted with post-traumatic stress and depression as independent variables, fear of progression as the mediating variable, and health-related quality of life as the latent dependent variable (Figure 1). Exploratory group comparisons and correlational analyses treating age continuously showed only subtle or negligible associations with psychosocial variables (a weak correlation was observed only with depression). Therefore, age was not included as a covariate, as it was unlikely to meaningfully affect the mediation effects.

The evaluation of the structural equation model parameters was performed using maximum likelihood and bootstrap methods, allowing the significance of the mediating effects to be tested. The goodness-of-fit indices indicated a good model fit: χ^2^(11) = 18.79; *p* = 0.065; χ^2^/df = 1.71; RMSEA = 0.078; 90 CI (0.000–0.136); *p* = 0.202; CFI = 0.978; and SRMR = 0.033. The bias-corrected percentile bootstrap analysis revealed a significant indirect effect of fear of progression on the relationship between post-traumatic stress and health-related quality of life [indirect effect 1; estimated effect = −0.174; 95% CI [−0.293, −0.084]; *p* < 0.001]. Another mediation effect of fear of progression was found in the relationship between depression and health-related quality of life [indirect effect 2; estimated effect = −0.181; 95% CI [−0.301, −0.086]; *p* < 0.001].

## 4. Discussion

This study aimed to analyze indicators of psychosocial adjustment in middle-aged and elderly women with a cancer diagnosis and to explore whether fear of progression (FoP) mediated the relationship between emotional distress, post-traumatic stress, and health-related quality of life (HQoL). In the exploratory analysis of sample characteristics, results showed that middle-aged and older women presented largely similar psychosocial profiles, with only subtle differences emerging between groups.

Older women reported slightly higher levels of depression and poorer physical and social functioning; however, these effects were small and may not reflect clinically meaningful distinctions. This pattern is not entirely surprising, as previous studies have already shown inconsistencies in this area. On one hand, some research suggests that middle-aged adults demonstrate a propensity to report higher levels of depression due to the accumulation of developmental demands, such as maintaining employment, caring for family, and sustaining social roles [43,44]. On the other hand, other studies report that older adults with chronic illnesses are more vulnerable to developing depressive symptoms [45], which is consistent with evidence showing a higher risk of comorbidities and mobility difficulties in this subgroup, and therefore a higher depression burden [46]. Additionally, depressive symptoms in the present sample were generally low, which may influence these findings and warrants cautious interpretation.

Similarly, the absence of differences in FoP, anxiety, and post-traumatic stress also contrasts with studies reporting worse outcomes among middle-aged adults [47,48], patterns that were not confirmed in the present study. These results are consistent with the view that FoP may reflect a more general vulnerability process that is relatively independent of age [20]. Other explanations may also be considered. Lim and Humphris [19], while noting an inverse relationship between age and FoP, found that the magnitude of this effect has decreased in more recent studies. This suggests a reduction in the disparity between FoP levels reported by younger and older patients compared to studies from the early 2010s. The authors propose that younger patients in older studies often reported higher FoP due to concerns about fulfilling family responsibilities. With increasing life expectancy, however, older adults may now perceive themselves as having more years ahead, making the threat of disease progression equally salient. In short, increased life expectancy may help explain the similarities observed across age groups.

Regarding anxiety, Stark and colleagues [49] concluded that although anxiety prevalence is significantly higher among individuals diagnosed with cancer, age has minimal impact on anxiety disorder estimates and is not a significant predictor. They argue that demographic differences that predispose individuals to anxiety in the general population become less relevant when facing a major life-threatening event such as cancer. Although no recent studies were found examining the association between age and post-traumatic stress, a 1998 study on breast cancer survivors found no association between age at diagnosis and post-traumatic stress symptoms [50]. Once again, this suggests that demographic variables such as age may be less relevant in the context of highly disruptive events. Comparisons across studies remain difficult due to methodological heterogeneity, including differences in age stratification, age at diagnosis, time since treatment, cancer type, and treatment modalities.

Given this pattern, age did not appear to function as a meaningful differentiating factor in emotional distress, FoP, post-traumatic stress, or HQoL. Preliminary correlational analyses reinforced this interpretation, justifying the decision to treat middle-aged and older women as a single analytic group in the subsequent model. Importantly, this decision is also consistent with the broader literature, where adults over 45 are frequently conceptualized as a later-adulthood group and remain markedly underrepresented in psychosocial research.

Based on this rationale, a path model was used to explore psychosocial adjustment in the context of oncological disease, considering the total sample.

The preliminary correlation analyses further emphasized the centrality of FoP, which showed robust positive associations with post-traumatic stress and moderate associations with depressive symptomatology.

These findings align with previous research suggesting that heightened FoP has been associated with higher distress levels and contributes to the persistence of trauma-related symptoms [20,51,52]. Additionally, it showed robust negative correlations with emotional, physical, and social functioning, demonstrating its close association with multiple domains of HRQoL Comparable results were observed by Simard and colleagues, who found that FoP predicted quality of life and functioning [53]. Crist and colleagues also found that physical symptoms were associated with or predicted high FoP, suggesting that survivors may attribute physical sensations to cancer recurrence or progression, or alternatively, that FoP increases symptom awareness. The direction of causality remains unclear [51].

Path analysis has provided new insights into the mediating role of FoP, complementing the existing literature on psychosocial adjustment to cancer. The indirect association observed between post-traumatic stress and HRQoL, through FoP, suggests that high levels of traumatic symptoms tend to coexist with heightened concerns about health trajectory, a pattern that is related to lower well-being. Similarly, the statistical mediating effect identified in the relationship between depression and HRQoL reinforces the role of FoP as an associated mechanism through which depressive symptomatology is linked to poorer functioning and a reduced perception of health. It is imperative, however, to interpret these mediations with caution: given the cross-sectional nature of the data, these trajectories describe the correlational structure between the constructs and not a temporal sequence of events or a direct causal influence.

These findings are of considerable interest, as they provide the first evidence of FoP’s mediating role in this age group. However, replication studies are needed to validate these results. Notwithstanding the contributions of this research, certain limitations must be acknowledged when interpreting the findings. First, the decision to focus exclusively on women was primarily methodological rather than based on an expectation that FoP operates differently by gender. Restricting the sample to women reduced heterogeneity associated with gender-specific patterns of emotional expression, caregiving roles, and cancer types, thereby allowing a more precise examination of the mechanisms linking fear of progression, psychological distress, post-traumatic stress, and health-related quality of life. Nonetheless, this choice limits the generalizability of the findings to male or mixed-gender populations, and future research should examine whether these mechanisms operate similarly across genders. Second, breast cancer was the most prevalent diagnosis, and the number of older participants was relatively small, which may further limit the generalizability of the results. Furthermore, future research should prioritize the recruitment of larger and more diagnostically diverse samples to enhance the external validity of the findings. Additionally, it is important to note that the study’s cross-sectional design precludes the ability to draw definitive causal inferences regarding the relationships between the variables. Although the mediation model provides insight into potential mechanisms, longitudinal studies are needed to examine the temporal dynamics of FoP and its impact on psychosocial adjustment. Moreover, more age-sensitive self-report measures may be useful in future research to enhance clinical utility, specificity, and sensitivity, particularly for distress symptoms [51]. Finally, the reliance on online recruitment strategies may have introduced self-selection bias. This approach potentially over-represents participants with higher levels of digital literacy or those more predisposed to share their clinical experiences, thereby constraining the representativeness and generalizability of the results to the broader cancer survivor population.

Nevertheless, the findings of this study have important clinical implications. Interventions targeting FoP may be beneficial in promoting better adjustment among cancer survivors. Furthermore, healthcare providers should consider screening FoP in routine clinical assessments to identify individuals at higher risk of HQoL deterioration.

## 5. Conclusions

This is one of the first studies to explore the role of fear of progression (FoP) in the psychosocial adjustment of middle-aged and older women with a cancer diagnosis. The results suggest that FoP is a mediator of the relationship between depression and post-traumatic stress and health-related quality of life. Our findings may have important implications for clinical settings, as they highlight the need for healthcare professionals to assess FoP in this population. By identifying individuals with higher levels of FoP, it may be possible to implement interventions that promote better adjustment and quality of life. These interventions may include psychoeducation about the disease and its treatments, cognitive–behavioral therapy to address FoP-related thoughts and behaviors, and relaxation techniques to manage anxiety. Future research is needed to validate the present findings and to develop and test the efficacy of interventions specifically targeting FoP in middle-aged and older women with an oncological diagnosis.

## Figures and Tables

**Table 1:** Sample description.

	Total Sample (*n* = 135)	Middle-Aged Adults (*n* = 109)	Older Adults (*n* = 26)
Variable	*n* (%)	*n* (%)	*n* (%)
Age in years (M ± DP; range)	52.94 ± 6.58; 45–79	50.39 ± 3.81; 45–59	63.62 ± 4.78; 60–79
Education			
Elementary Education	26 (19.3)	17 (15.6)	9 (34.6)
Secondary Education	36 (26.7)	32 (29.4)	4 (15.4)
Undergraduate Degree	58 (43.0)	47 (43.1)	11 (42.3)
Master’s Degree	10 (7.4)	8 (7.3)	2 (7.7)
Doctoral Degree (PhD)	3 (2.2)	3 (2.8)	0 (0)
Marital status			
Single	9 (6.7)	8 (7.3)	1 (3.8)
Married/Cohabiting	93 (68.9)	75 (68.9)	18 (69.2)
Divorced/Separated	29 (21.5)	23 (21.1)	6 (23.1)
Widowed	4 (3.0)	3 (2.8)	1 (3.8)
Employment status			
Employed/Self-employed	61 (45.2)	55 (50.5)	6 (23.1)
Unemployed	15 (11.1)	11 (10.1)	4 (15.4)
Household/Domestic Worker	6 (4.4)	6 (5.5)	0 (0)
Temporary Work Disability	23 (17.0)	21 (19.3)	2 (7.7)
Retired	25 (18.5)	12 (11.0)	13 (50.0)
Household composition			
Lives Alone	21 (15.6)	13 (11.9)	8 (30.8)
Lives with Spouse/Partner	92 (68.1)	74 (67.9)	18 (69.2)
Lives with Son/Daughter	82 (60.7)	75 (68.8)	7 (26.9)
Lives with Father/Mother	1 (0.7)	1 (0.9)	0 (0)
Current stage of the cancer treatment			
Undergoing Treatment	53 (39.3)	40 (36.7)	13 (50.0)
Remission	82 (60.7)	69 (63.3)	13 (50.0)
Cancer type			
Breast	105 (77.8)	87 (79.8)	18 (69.2)
Colorectal	5 (3.7)	4 (3.7)	1 (3.8)
Lung	5 (3.7)	5 (4.6)	0 (0)
Kidney	2 (1.5)	2 (1.8)	0 (0)
Stomach	2 (1.5)	2 (1.8)	0 (0)
Lymphoma	2 (1.5)	0 (0)	2 (7.7)
Sarcoma	1 (0.7)	1 (0.9)	0 (0)
Other	13 (9.6)	8 (7.3)	5 (19.2)
Time since initial diagnosis in years (M ± DP; range)	4.24 ± 4.02; 0.58–28.6	3.86 ± 3.68; 0.58–28.6	5.81 ± 4.99; 0.58–16.8
Previous treatments			
Chemotherapy	103 (76.3)	80 (73.4)	23 (88.5)
Radiation Therapy	83 (61.5)	67 (61.5)	16 (61.5)
Hormone Therapy	86 (63.7)	69 (63.3)	17 (65.4)
Immunotherapy	27 (20.0)	23 (21.1)	4 (15.4)
Surgery	99 (73.3)	81 (74.3)	18 (69.2)
Other clinical conditions			
Diabetes	3 (2.2)	2 (1.8)	1 (3.8)
Hypertension	18 (13.3)	15 (13.8)	3 (11.5)
Cholesterol	14 (10.4)	7 (6.4)	7 (26.9)
Obesity	12 (8.9)	10 (9.2)	2 (7.7)
Respiratory Diseases	9 (6.6)	4 (3.7)	5 (19.1)
Osteoarthritis	13 (9.6)	10 (9.2)	3 (11.5)
Osteoporosis	12 (8.9)	7 (6.4)	5 (19.2)
Use of mental health services	51 (37.8)	40 (36.7)	11 (42.3)

**Table 2:** Comparing psychosocial adjustment indicators between middle-aged and older women.

	Middle-Aged Adults (*n* = 109)	Older Adults (*n* = 26)	U	Effect Size
Variable	Mdn|M|IQR	Mdn|M|IQR		
Fear of Progression	40|40.7|15.5	42.5|42.9|12.8	1212.5	0.1
Anxiety	9|8.6|7	10|9.6|5	1201	0.1
Depression	6|5.9|5	7|7.6|3.25	1038 *	0.2
Post-Traumatic Stress (total score)	2.7|2.7|1.53	2.9|2.9|1.19	978.5	0.1
Re-experience	2.4|2.7|2	3.2|3.0|1.4	858.5	0.1
Avoidance	2.4|2.5|1.86	2.9|2.7|1.71	976.5	0.1
Hyperactivation	3.0|3.0|1.8	2.8|2.8|1.3	911.5	0.1
Emotional Functioning	50|51|33.3	41.7|41.7|41.67	1083	0.2
Physical Functioning	73.3|71.9|33.3	70|61.3|40	988 *	0.2
Role Functioning	66.7|63.4|33.3	58.3|53.2|33.3	1098.5	0.2
Social Functioning	50|49.4|33.3	33.3|37.8|33.3	1014 *	0.2

* *p* < 0.05; Mdn = median; M = mean; IQR = interquartile range; U = Mann–Whitney U test.

**Table 3:** Bivariate correlations: relationships between age, fear of progression, psychological distress, post-traumatic stress, and quality of life in cancer survivors.

	1.	2.	3.	4.	5.	6.	7.	8.	9.	10.	11.	12.
1. Fear of Progression												
2. Anxiety	0.734 ***											
3. Depression	0.489 ***	0.649 ***										
4. Post-Traumatic Stress	0.508 ***	0.499 ***	0.389 ***									
5. Re-experience	0.466 ***	0.424 ***	0.338 ***	0.902 ***								
6. Avoidance	0.437 ***	0.437 ***	0.419 ***	0.911 ***	0.720 ***							
7. Hyperactivation	0.471 ***	0.474 ***	0.259 ***	0.886 ***	0.741 ***	0.705 ***						
8. Emotional Functioning	−0.605 ***	−0.600 ***	−0.499 ***	−0.358 ***	−0.302 ***	−0.251 **	−0.416 ***					
9. Physical Functioning	−0.528 ***	−0.428 ***	−0.474 ***	−0.329 ***	−0.305 ****	−0.270 **	−0.313 ***	0.440 ***				
10. Role Functioning	−0.429 ***	−0.396 ***	−0.384 ***	−0.298 ***	−0.329 ***	−0.174	−0.309 ***	0.570 ***	0.518 ***			
11. Social Functioning	−0.593 ***	−0.464 ***	−0.457 ***	−0.442 ***	−0.402 ***	−0.369 ***	−0.414 ***	0.679 ***	0.536 ***	0.506 ***		
12. Age (continuous)	0.033	0.050	0.216 *	0.030	0.088	0.081	−0.133	−0.091	−0.157	−0.140	−0.042	_

* *p* < 0.05; ** *p* < 0.01; *** *p* < 0.001.

## Data Availability

The data that support the findings of this study are available from the corresponding author [S.S.] upon reasonable request.
